# The *hrpZ* Gene of *Pseudomonas syringae* pv. *phaseolicola* Enhances Resistance to Rhizomania Disease in Transgenic *Nicotiana benthamiana* and Sugar Beet

**DOI:** 10.1371/journal.pone.0017306

**Published:** 2011-03-04

**Authors:** Ourania I. Pavli, Georgia I. Kelaidi, Anastasia P. Tampakaki, George N. Skaracis

**Affiliations:** 1 Department of Crop Sciences, Agricultural University of Athens, Athens, Greece; 2 Department of Agricultural Biotechnology, Agricultural University of Athens, Athens, Greece; Massachusetts Institute of Technology, United States of America

## Abstract

To explore possible sources of transgenic resistance to the rhizomania-causing *Beet necrotic yellow vein virus* (BNYVV), *Nicotiana benthamiana* plants were constructed to express the harpin of *Pseudomonas syringae* pv. *phaseolicola* (HrpZ*_Psph_*). The HrpZ protein was expressed as an N-terminal fusion to the PR1 signal peptide (SP/HrpZ) to direct harpin accumulation to the plant apoplast. Transgene integration was verified by mPCR in all primary transformants (T0), while immunoblot analysis confirmed that the protein HrpZ*_Psph_* was produced and the signal peptide was properly processed. Neither T0 plants nor selfed progeny (T1) showed macroscopically visible necrosis or any other macroscopic phenotypes. However, plants expressing the SP/HrpZ*_Psph_* showed increased vigor and grew faster in comparison with non-transgenic control plants. Transgenic resistance was assessed after challenge inoculation with BNYVV on T1 progeny by scoring of disease symptoms and by DAS-ELISA at 20 and 30 dpi. Transgenic and control lines showed significant differences in terms of the number of plants that became infected, the timing of infection and the disease symptoms displayed. Plants expressing the SP/HrpZ*_Psph_* developed localized leaf necrosis in the infection area and had enhanced resistance upon challenge with BNYVV. In order to evaluate the SP/HrpZ-based resistance in the sugar beet host, *A. rhizogenes*-mediated root transformation was exploited as a transgene expression platform. Upon BNYVV inoculation, transgenic sugar beet hairy roots showed high level of BNYVV resistance. In contrast, the aerial non-transgenic parts of the same seedlings had virus titers that were comparable to those of the seedlings that were untransformed or transformed with wild type R1000 cells. These findings indicate that the transgenically expressed SP/HrpZ protein results in enhanced rhizomania resistance both in a model plant and sugar beet, the natural host of BNYVV. Possible molecular mechanisms underlying the enhanced resistance and plant growth phenotypes observed in SP/HrpZ transgenic plants are discussed.

## Introduction

Rhizomania disease of sugar beet is responsible for a very significant reduction in crop's productivity globally, as a consequence of a considerable decrease in root yield, sugar content and juice purity [1], [2]. Since the initial reports of the disease more than half a century ago, its causal agent *Beet necrotic yellow vein virus* (BNYVV) has spread to all major sugar beet growing countries [2]. Given the absence of other control strategies, the only substantial means to ensure a viable crop production in rhizomania infested areas is the use of varieties specifically bred for resistance to the virus [3]. In this respect, coping with rhizomania to date has been based mainly on cultivars endowed with the *Rz1* resistance gene (“Holly” source), a dominant gene conferring sufficiently high levels of protection against BNYVV [4], [5]. In addition to conventional breeding methodologies that led to all currently rhizomania resistant sugar beet varieties, various genetic engineering approaches have also been studied for the purpose of enhancing disease resistance. These include pathogen-derived resistance (PDR), relying on the transgenic expression of viral genes/sequences [6], [7], antibody-mediated resistance [8] and RNA silencing-mediated resistance, the most successful variant of PDR [9]–[11]. However, recent changes in the field and molecular BNYVV epidemiology, as manifested by the emergence of type-A virus strains capable of compromising the *Rz1-*based resistance [12], [13] and the spread of highly pathogenic type P-isolates [14], necessitate further research for alternative forms of resistance against BNYVV.

Harpins constitute a class of phytobacterial Type III Secretion System (T3SS) components that are readily secreted by bacteria in culture media and are thought to act as accessory proteins for effector translocation during the host-pathogen interaction [15]. Harpins have been described in several plant pathogenic bacteria including members of the genera *Erwinia*, *Pantoea*, *Pseudomonas*, *Xanthomonas* and *Ralstonia* [16]–[24]. Although their primary sequences are rather divergent, harpins share the following characteristics: they are glycine-rich, cysteine-free, heat stable, acidic, protease-sensitive and, in contrast to other T3SS effectors, they are capable of triggering plant responses such as the hypersensitive response (HR) when infiltrated in a purified form into the leaf apoplast. In addition, harpins induce a series of molecular/cellular-level responses associated with resistance to various pathogens, either when externally applied to plants or produced endogenously after stable transformation or transient expression. These defense responses are either local or, often, systemic leading to the development of systemic acquired resistance (SAR) at the whole plant level [25]–[27]. Apart from promoting defense-related functions, harpins also influence the regulation of plant growth, presumably by enhancing nutrient uptake and increasing photosynthesis [28]–[30].

The demonstration that phytobacterial harpins are capable of eliciting HR and/or stimulating defense gene expression led to their exploitation as phytoprotectants against bacteria, fungi, viruses, insects, as well as against abiotic stresses [25], [26], [28], [31]–[37]. Although sharing common characteristics, phytobacterial harpins are not very similar in primary sequence and therefore not all plants recognize all harpins. Thus, the efficient use of harpins as phytoprotectants raises the question as to which harpin is more appropriate in a particular plant species. Another important consideration is the site of harpin accumulation and site(s) of harpin action in the plant cell. Several lines of evidence indicate that harpins act extracellularly. First, several studies indicate that harpins may interact with extracellularly exposed components of plant cells [30], [38], [39]. Second, some harpins form ion-conduction pores in artificial membrane bilayers and insert themselves into the plant plasma membranes forming pores, ostensibly to facilitate the T3SS translocation process i.e. the entry of effector proteins into plant cells [40]–[42]. The latter activity is structurally separable from that of triggering the plant immune response [43]. However, recent studies showed that small amounts of the *Erwinia amylovora* (HrpN) and *P. syringae* pv. *tomato* DC3000 (HrpZ1) harpins are capable of being translocated into plant cells of tobacco leaves by the bacterial T3SS [42], [44]. Thus, whether or not the primary site of harpin action in plant cells is extra- or intracellular remains to be elucidated. Taken together, our knowledge of the physiological and molecular effects as well as the actual site of action of a particular harpin *in planta* may have an impact on genetic engineering strategies that aim to increase plant resistance.

In this study, the potential of HrpZ*_Psph_*, the harpin protein from *Pseudomonas syringae* pv. *phaseolicola* in conferring transgenic resistance to the rhizomania-causing BNYVV has been explored. A secretable form of HrpZ*_Psph_* (SP/HrpZ*_Psph_*) was expressed in stable transformants of *Nicotiana benthamiana* plants and in transgenic sugar beet hairy roots to investigate its effects on virus titer and symptoms following BNYVV inoculation.

## Materials and Methods

### Bacterial strains and plasmids

*Agrobacterium tumefaciens* strain C58C1 (Rif^R^) carrying the binary plant expression vector construct pBin.Hyg.Tx-*SP/hrpZ_Psph_* [39] was used to transform *N. benthamiana* plants. The pBin.Hyg.Tx-*SP/hrpZ_Psph_* construct carries the *hrpZ* gene from *P. syringae* pv. *phaseolicola* NPS3121 (approx. 1 kb) cloned downstream of the CaMV35S promoter. The *hrpZ* coding region is fused in-frame with region coding for the signal peptide from the tobacco pathogenesis-related protein PR1 [39]. Bacterial cells were grown at 28°C in liquid LB medium containing rifampicin (50 µg ml^−1^), carbenicillin (100 µg ml^−1^) and kanamycin (50 µg ml^−1^) for 2 days or until OD_600_ = 0.6–1.0 was reached. Following centrifugation, bacterial cells were resuspended in MS to a final concentration of 10^8^ cfu/ml and the cell suspension was used as inoculum for plant transformation.

For transformation of sugar beet roots, the *A. rhizogenes* strain R1000 harbouring the plasmid pRiA4 was used. The pBin.Hyg.Tx-*SP/hrpZ_Psph_* construct was introduced to *A. rhizogenes* cells by electroporation and cultures were grown at 28°C under nalidixic acid (25 µg ml^−1^) and kanamycin (50 µg ml^−1^) selection until OD_600_ = 0.6–1.0 was reached. Bacterial cells were collected by centrifugation and the pellet was used as inoculum for plant transformation.

### Plant transformation and molecular characterization

Leaf discs from 5–6 week-old healthy plants of *N. benthamiana* were transformed using a standard protocol [45]. Selection of primary transformants was performed on the basis of resistance to hygromycin (30 µg ml^−1^). Regenerated shoots were subsequently rooted and transferred to soil. The presence of the transgene and absence of disarmed Ti plasmid sequences in the regenerated plants were confirmed by means of a multiplex PCR assay, using specific primers (Table 1) to amplify the 995 and 590 bp segments of *hrpZ_Psph_* and *virG* of *A. tumefaciens* respectively. Plants that were PCR-positive for the transgene and negative for *vir* genes were selfed and progeny (T1) were germinated on selective MS medium containing hygromycin (30 µg ml^−1^). Using standard tissue culture procedures for shoot and root formation, hygromycin-resistant seedlings were grown *in vitro* before being transferred to pots.

**Table 1:** Primers used in the present study for the amplification of transgene and defense-related genes.

Primer	Sequence (5′→3′)	Product size (bp)
hrpZ_Psph_-F	CGAAAGCCCGCATATGGCGCTCGTTCTG	995
hrpZ_Psph_-R	CCGTCAGCGGGATCCAGTCAGGCAGCAG	
virG-F	GCCGGGGCGAGACCATAGG	590
virG-R	CGCACGC-GCAAGGCAACC	
AOX-F	GCCATTGATTACTGCCGTCT	160
AOX-R	ATACCCAATTGGTGCTGGAG	
Col1-F	CCAATTGGGCTTGACGTACT	228
Col1-R	CAATCCTGAGCCGCTTTAAC	
EF1a-F	GAGGTTCGAGAAG-GAAGCTGCTGAG	669
EF1a-R	AGAGCTTCGTGGAGCATCTCAACAG	
Hin1-F	GAGCTCTAGATGGCCCTTCCATTCCGC	847
Hin1-R	GCTCTAG-ACGCCGGAAAAACAAAAGG	
Hsr203J-F	CGCGGATCCGGCTGGCTTAGAGTTTTC	596
Hsr203J-R	TCCGGGATCCTCCGATAGGACCGCACG	
NPR1-F	ATGGATAATAGTAGGACTGCG	273
NPR1-R	GAACGGACTCCTCGCCGAC	
PR1a-F	GTAATATCCCACTCTTGCCGTGCCC	335
PR1a-R	CCTAGCACATCCAACACGAACCGAG	
SIPK-F	TATAATTCCACCACCACAGA	755
SIPK-R	CTTCATCTGTTCCTCCGTAA	
WIPK-F	CAATTCCCTGATTTTCCTTCGG	1158
WIPK-R	GGAAAGTAGATACTCCAGATC	

The generation of sugar beet seedlings with a transgenic hairy root system was performed according to the *A. rhizogenes*-mediated transformation method described by Pavli and Skaracis [46]. Hygromycin-resistant sugar beet roots were evaluated for the presence of the cassette and for absence of *A. rhizogenes* cells using a multiplex PCR assay targeting the nucleotide sequences of the *hrpZ_Psph_* transgene and *virCD* of *A. rhizogenes*, using FTA (Whatman)-immobilized nucleic acids as template for amplification.

### Protein extraction and immunoblot analysis

Regenerated hygromycin-resistant plantlets were assayed for the accumulation of the *SP/hrpZ_Psph_* gene product by immunoblot analysis. Total soluble protein from lyophilized leaf/root material was extracted in SDS sample buffer. Following boiling for 5 min at 100°C, samples were separated on a 12% sodium dodecyl sulfate (SDS)-polyacrylamide gel and transferred to nitrocellulose membrane, using standard procedures. Immunoblotting was carried out using an anti-HrpZ*_Psph_* antibody at a 1∶20000 dilution [39]. The membrane was developed with an alkaline phosphatase-conjugated antibody with nitroblue tetrazolium chloride and 5-bromo-4-chloro-3-indolyl phosphate (NBT/BCIP), according to the supplier's instructions.

### Induction of defense-related genes

The induction of the HR- and defense-associated genes (*AOX*, *COI1*, *NPR1*, *SIPK*, *WIPK*, *hsr203J*, *PR1a*, *hin1*) due to harpin expression was examined by an RT-PCR assay. Total RNA was extracted using the SV Total RNA Isolation kit (Promega) and RNA samples were treated with RNase-free DNAse (Promega) prior to RT-PCR. Two µg of total RNA was used as template for synthesizing first-strand cDNA with oligo dT and reverse-transcribed using the Im-Prom II Reverse Transcriptase System (Promega), according to the supplier's instructions. Targeted sequences were amplified using the primer pairs described in Table 1. PCR reaction mixture contained 1 µl cDNA, 0.25 µM of each primer, 200 µM dNTPs, 1.25 mM MgCl_2_, 1× *Taq* buffer and 1.25 u *Taq* polymerase (GoTaq Flexi DNA polymerase, Promega) in a final volume of 25 µl. PCR conditions included initial denaturation at 94°C for 3 min, followed by different number of cycles at 94°C for 30 sec, 50°C for 1 min and 72°C for 1 min, then final elongation at 72°C for 7 min. PCR reactions for each gene were performed for 20, 22, 24, 26 and 28 cycles and the amplification products were analyzed on 1.0% agarose gels stained with ethidium bromide.

### Virus inoculations

BNYVV-infected sugar beet plants were used as virus source for inoculation of both transgenic *N. benthamiana* plants and sugar beet seedlings consisted of a transgenic root system. Based on previous characterization, sugar beets used for virus inoculations were infected with A-type BNYVV, with amino acid motif VCHG in the hypervariable region aa_67–70_ of RNA 3-encoded p25 [47].

A total of 12 transgenic *N. benthamiana* T0 lines were evaluated for virus resistance. In total, fifteen T1 plantlets from each transgenic line were mechanically inoculated and subsequently assessed visually as well as by DAS-ELISA. Foliar inoculations were performed by rubbing 3 carborundum dusted leaves with fresh inoculum prepared as described in Pavli et al. [11]. Non-transgenic *N. benthamiana* plants served as positive controls, whereas non-inoculated untransformed plants were included as experimental negative controls.

Evaluation of virus resistance in sugar beet was performed in two consecutive challenge-inoculation experiments by using a total of 24 transformed seedlings. Virus inoculation was performed according to the procedure described by Pavli et al. [11]. Seedlings transformed with wild type R1000 cells and untransformed seedlings were used as positive controls susceptible to BNYVV inoculation, while untransformed seedlings non-challenged with BNYVV served as negative controls.

### Assessment of virus resistance

For evaluation of disease resistance in transgenic *N. benthamiana*, plants were regularly monitored and symptoms were scored during the 30 day period post inoculation (dpi) while virus titers were determined at 20 and 30 dpi by means of DAS-ELISA (Adgen Phytodiagnostics) according to the supplier's instructions. Replicate leaf tissue samples were homogenized in 1∶3 or 1∶5 extraction buffer.

In sugar beet, estimation of virus titer was performed at 21 dpi, both in the transgenic hairy roots and in the non-transgenic aerial parts of the same seedlings. Plant sap from root and leaf tissue was separately extracted as earlier described and replicated twice in the plate. Absorbance values at 405 nm measuring at least three times the respective values of the negative controls were considered as positives.

## Results

### Generation of transgenic *N. benthamiana* plants expressing *SP/hrpZ_Psph_*

**Figure 1:**
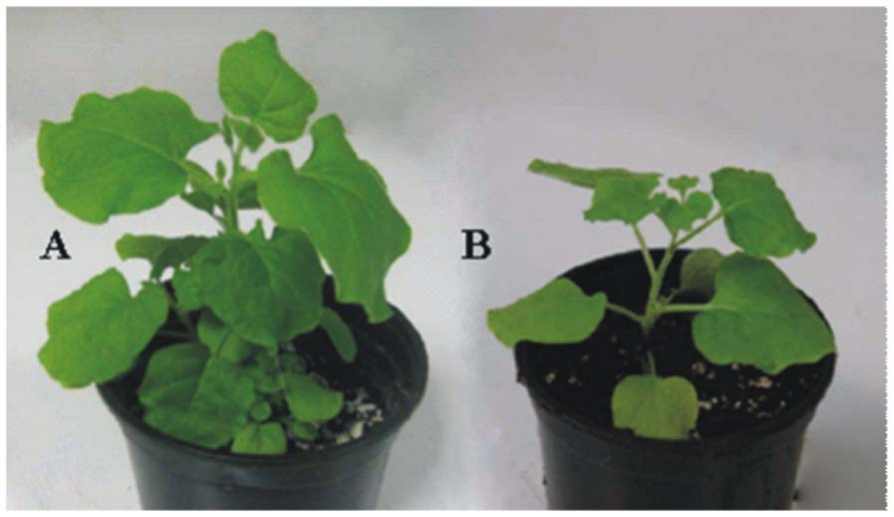
Promotion of plant growth due to harpin expression. A) A representative T1 transgenic *N. benthamiana* plant expressing SP/HrpZ*_Psph_*. B) Non-transgenic *N. benthamiana* plant of the same age.

Transgenic *N. benthamiana* plants constitutively expressing the secretable form of HrpZ*_Psph_* protein were constructed by *A. tumefaciens*-mediated leaf disc transformation with the plasmid pBin.Hyg.Tx-*SP/hrpZ_Psph_* [39]. Thirty nine independent transgenic lines (T0) were obtained and twelve of them were randomly selected and self-pollinated to produce T1 lines. Five T1 transgenic plants deriving from each of the twelve T0 lines were further analyzed. All primary transgenics (T0) and sixty selfed progeny (T1) showed no necrotic or other type of symptoms and generally developed a normal phenotype. However, all T0 and T1 transgenic plants showed increased vigor and a rapid growth rate. Although all transgenic plants tested shared similar enhanced growth patterns, these were significantly differentiated compared to the untransformed plants. Figure 1 shows the growth enhancement observed between a control untransformed plant and a representative T1 plant of the same age.

**Figure 2:**
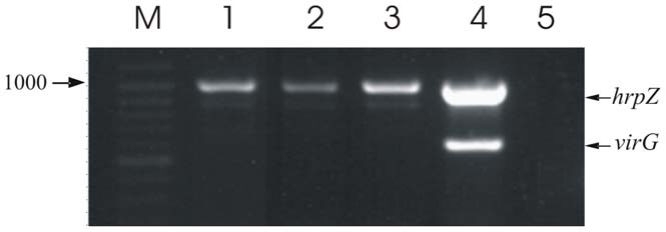
Amplification products obtained by multiplex PCR on total genomic DNA from *Agrobacterium*-transformed *N. benthamiana* plants. Lane M: Marker in bp (Gene Ruler Ladder mix, Fermentas). Lanes 1, 2, 3: Representative T1 transgenic plants, carrying the 995 bp fragment of *hrpZ_Psph_*. Lane 4: *A. tumefaciens* C58C1 cells carrying pBin.Hyg.Tx-*SP/hrpZ_Psph_*. Lane 5: untransformed control plant. The 590 bp amplicon corresponding to *virG* of *A. tumefaciens* could only be obtained using bacterial cells as a template, thus verifying the absence of Ti plasmid sequences in hygromycin resistant *N. benthamiana* transformants.

**Figure 3:**
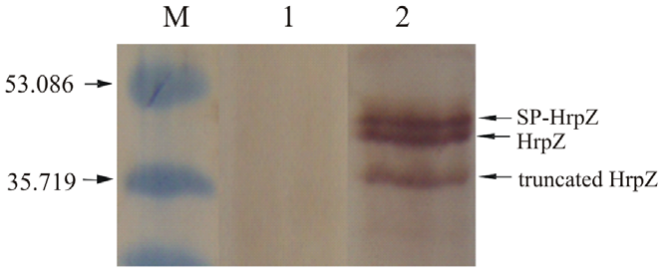
Western blot analysis of SP/HrpZ*_Psph_* in protein extracts from leaves of *N. benthamiana* plants. Lane M: Marker in kDa (Broad range pre-stained SDS marker, Biorad). Lane 1: Non-transgenic plant. Lane 2: T1 transgenic plant transformed with the plasmid pBin.Hyg.Tx-*SP/hrpZ_Psph_*. The lower band represents the truncated form of the harpin (approx. 2 kDa smaller than the full-length HrpZ*_Psph_*).

Multiplex PCR analysis targeting fragments of *hrpZ_Psph_* and *virG* of the disarmed Ti plasmid indicated transgene integration and absence of *A. tumefaciens* in all T1 and T0 lines tested. Figure 2 shows the presence of *hrpZ* amplicons in representative T1 transgenic plants while the absence of *virG* amplicons is indicative that transgenic plants are bacteria-free. Immunoblot analysis of all sixty T1 transgenic lines indicated that the protein was indeed produced and the signal peptide was properly processed as shown in Figure 3 for a representative T1 line. In transgenic plants, three immunoreactive bands were detected corresponding to SP/HrpZ, HrpZ and a truncated form of the protein. The latter is probably a product of internal initiation of translation in the ATG codon at position 16 of the harpin ORF in plant cells, as reported earlier [39]. The SP/HrpZ band presumably represents cytosolic protein “en route” to be secreted in the apoplast from which the signal peptide has not yet been cleaved.

### *SP/HrpZ_Psph_-*expressing *N. benthamiana* plants show enhanced resistance and localized necrosis in the inoculated leaf area

BNYVV inoculation of T1 progeny was carried out at the 5- to 6-leaf stage. Five progeny plants (T1), for each of the twelve transgenic *N. benthamiana* lines (T0), were evaluated visually and analyzed by DAS-ELISA for virus infection. Challenge inoculated experiments were repeated three times by choosing different T1 plants in each experiment. Totally, fifteen T1 plants for each of the twelve T0 lines (in total 180 T1 plants) were evaluated on the basis of BNYVV resistance.

Non-transgenic plants developed faint mosaic and leaf curling symptoms beginning at 10–14 dpi and displayed symptoms of severe mosaic, occasional leaf distortion and a general stunting at 20–22 dpi. These findings were positively correlated with virus titers as estimated by ELISA (Table 2).

**Table 2:** Symptom severity and virus titer of transgenic *N. benthamiana* T1 plants, 30 days after challenge with BNYVV.

Evaluation of resistance (T1 plants)						
Type of plants	T1 plants tested	T0 origin	1Symptom development		2Virus titer	
SP/HrpZ*_Psph_* *	180	7	−105^3^	(58.3%)^4^	−105	(58.3%)
		5	−47	(26.1%)	−19+28	(10.6%)(15.6%)
			+23	(12.8%)	+17++6	(9.4%)(3.3%)
			++5	(2.8%)	++5	(2.8%)
**Non-transgenic**	30		+++30	(100%)^3^	+++30	(100%)

1 − absence of symptoms, + leaf curling, ++ faint mosaic, mild stunting, +++ severe mosaic, leaf distortion, general stunting.

2 − value indicative of virus absence, + close to the positive threshold (three times the negative control), ++ half reading of positive control (non-transgenic), +++ equal to the positive control.

3 numbers denote number of plants in corresponding categories.

4 numbers in parentheses denote percent of plants in corresponding categories.

*Data in the column refer to the averages from four independent experiments used as validation to the third column.

Based on symptom severity, all T1 *SP/hrpZ_Psph_*-expressing plants tested were characterized as highly resistant to infection. The majority of them (84.4%) remained completely symptomless throughout the period of observation. The remaining T1 plants (15.6%) showed delayed symptom development by at least 12–14 days compared to the non-transgenic ones (Table 2). More specifically, all the T1 plants deriving from seven of the twelve T0 lines were entirely symptomless and virus-free based on visual scoring and ELISA test respectively (58.3%). The T1 progeny of the remaining five T0 lines were also highly resistant, a significant portion (47 plants) remaining symptomless (62.5%), while in the rest of plants (28 plants) (37.5%), the disease symptoms were mild and virus titers were relatively low. Among the symptomless plants, 40% of them proved completely virus-free (19 plants). At 20 dpi, nearly all transgenic plants (91.4%) were negative for BNYVV accumulation as determined by ELISA (data not shown). At 30 dpi, 68.9% of *SP/hrpZ_Psph_*-expressing plants still remained essentially virus-free while 31.1% had a virus titer that was very low or near the scoring threshold. These findings demonstrate that the production of the *P. syringae* pv. *phaseolicola* harpin in its secretable form in *N. benthamiana* results in high-level resistance to BNYVV.

**Figure 4:**
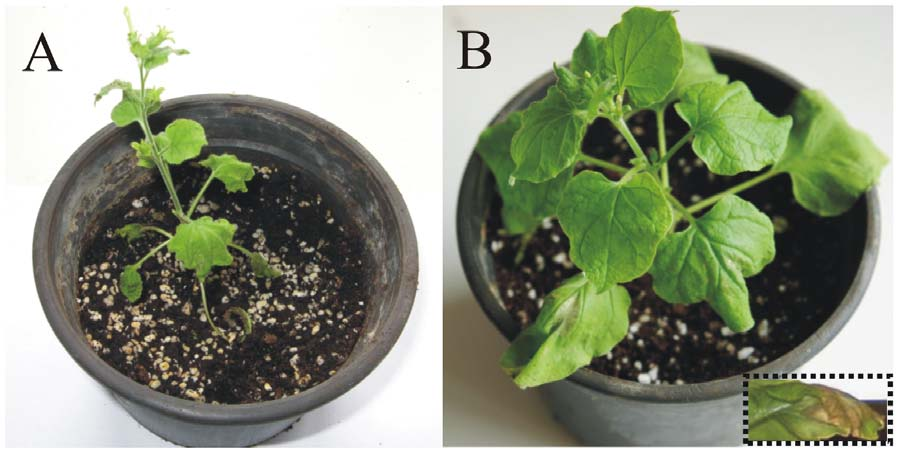
Induction of tissue necrosis in the BNYVV inoculated leaf area of transgenic *N. benthamiana* plant lines expressing SP/HrpZ*_Psph_*. A) Non-transgenic plant. B) SP/HrpZ*_Psph_*-expressing plant. The inset shows the tissue necrosis localized in the inoculated leaf area of a representative resistant T1 plant at 3–4 dpi.

Interestingly, BNYVV inoculation of the *SP/hrpZ_Psph_*-expressing plants was followed by conspicuous tissue necrosis, localized exclusively in the inoculated leaf area, at 3–4 dpi (Figure 4). The appearance of this necrosis was characteristic of only transgenic lines and was highly consistent among all plants tested. A possible interpretation of this phenotype is discussed below.

### Defense-associated gene activation in harpin-expressing plants

Previous studies have shown that harpins activate several plant defense pathways. It has also been reported that different defense-related genes are induced by either different harpins or different ways of applications i.e. exogenous or endogenous. To investigate the correlation between the BNYVV resistance developed in transgenic plants and the expression level of several defense-associated genes, we carried out semi-quantitative RT-PCR using total RNA from leaves of a non-transgenic, three highly resistant T1 transgenic plants expressing the secretable form of harpin which were completely symptomless and virus-free as well as the non-secretable one, before inoculation with BNYVV.

We examined the expression of *hin1*, *hsr203J*, *SIPK*, *WIPK*, which are HR-associated genes, and *AOX*, *COI1*, *NPR1* and *PR1a*, which are involved in active oxygen species (AOS)-, jasmonic acid (JA)-, salicylate (SA)- and PR-dependent defense pathways, respectively [48]–[53].

**Figure 5:**
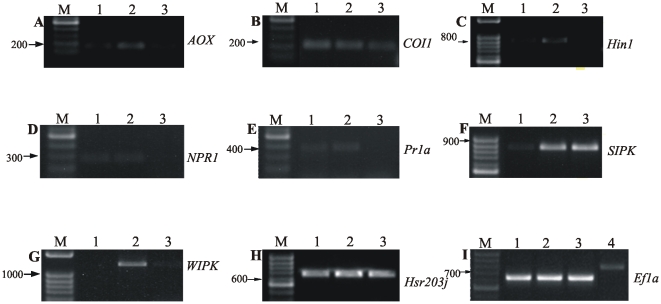
Induction of defense-related genes in harpin-expressing plants. Expression of *AOX* (A), *COI1* (B), *hin1* (C), *NPR1* (D) *PR1a* (E), *SIPK* (F), *WIPK* (G), *hsr203J* (H) and *EF1a* (I) determined by semi-quantitative RT-PCR in *hrpZ_Psph_*- (1), *SP/hrpZ* ***_Psph_*** **-**transgenic (2) and wild-type (3) *N. benthamiana* plants. Lane M: Marker in bp (100 bp DNA Ladder, NIPPON Genetics). Lane 4: Genomic DNA from leaves of wild-type *N. benthamiana* plants, used as template to assess possible DNA contamination in the RNA extracts from a transgenic plant: the amplicon corresponding to *EF1a* gene is of larger size in comparison with the transcript obtained by RT-PCR. Amplicons corresponding to the *AOX* (160 bp), *COI1* (228 bp), *hin1* (847 bp), *NPR1* (273 bp), *PR1a* (335 bp), *SIPK* (755 bp), *WIPK* (1158 bp), *hsr203J* (596 bp) and EF1a genes (669 bp) at 26, 28, 22, 28, 28, 24, 24, 22 and 30 amplification cycles respectively.

*SIPK*, *hsr203J* and *COI1* were expressed at similar levels in transgenic and non-transgenic plants tested, suggesting that these genes might not be involved in the development of harpin-induced resistance against BNYVV (Figure 5). The expression level of *AOX*, *NPR1* and *PR1a* amplicons was slightly higher in the transgenic plants compared to the non-transformed one. On the other hand, the expression of *WIPK* and *hin1* was significantly higher in all *SP-hrpZ_Psph_* transgenic plants tested than in *hrpZ_Psph_* transgenic and control non-transgenic plants.

To rule out the possibility that the amplification of these sequences was due to a contamination by genomic DNA, we used the constitutively expressed *EF1a* gene as internal control. The *EF1a*-amplicon obtained from genomic DNA is of larger size-due to the presence of an intron- compared to that of the transcript obtained by RT-PCR from leaf mRNA extracts (Figure 5).

### The expression of SP/HrpZ_Psph_ confers high level of BNYVV resistance in transgenic sugar beet hairy roots

In order to confirm the SP/HrpZ_Psph_-based resistance in the sugar beet host, the *A. rhizogenes* hairy root system [46] was exploited as a suitable platform for the expression of the harpin protein. PCR analysis targeting fragments of *hrpZ_Psph_* and *virCD* indicated transgene integration and absence of *A. rhizogenes* in all roots tested (data not shown). In addition, *hrpZ_Psph_* transgene expression was verified by western blot analysis in all root samples examined (data not shown). Transformed seedlings were subsequently evaluated for virus resistance by means of visual assessment and ELISA-mediated measurement of virus titer at 21 dpi. In total, 24 transformed seedlings were included in two challenge-inoculation experiments.

**Figure 6:**
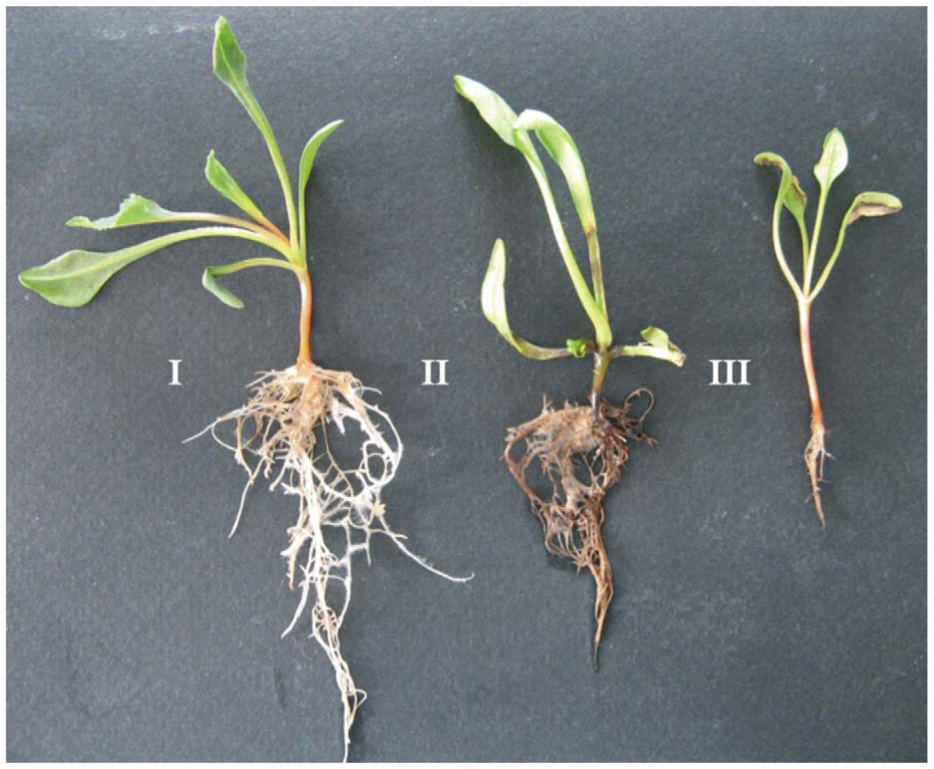
Symptoms of *Beet necrotic yellow vein virus* in sugar beet seedlings at 21 days post inoculation. I) Seedling with a *SP/hrpZ_Psph_*-expressing transgenic root system. II) Seedling transformed with wild type R1000 cells. III) Untransformed seedling with a wild type root system. Transgenic roots expressing the protein are symptomless whereas control seedlings, exhibit symptoms of root deterioration as well as leaf chlorosis and necrosis.

Upon BNYVV inoculation, all positive control seedlings i.e. untransformed and transformed with wild type R1000 strain were severely infected and developed a severe stunting, leaf curling and localized necrosis at 21 dpi (Figure 6). In general, ELISA values were well correlated with these phenotypic observations. As expected, the root system of these plants consistently showed higher virus titers (average OD_405_ = 0.794) than the aerial part of the same seedlings (average OD_405_ = 0.627) (Table 3).

**Table 3:** Mean of BNYVV ELISA values of two challenge-inoculation experiments.

ELISA Readings (OD_405_)			
Type of seedlings	Number of seedlings tested	21 dpi	
		Aerial part	Root system
SP-HrpZ_Psph_	24	0.492 a B*	0.148 b B
Positive control**	12	0.627 b A	0.794 a A
Negative control	12	0.112	0.128

Data represent the average OD_405_ readings of each seedling type at 21 days post inoculation (dpi).

*Different letters denote statistically significant differences at *P = 0.05*. Small letters refer to horizontal comparisons between roots and leaves (paired t-test). Capital letters refer to comparisons among the two categories of means (*SP-hrpZ_Psph_*-expressing and control seedlings) (F-test).

**Average of untransformed and wild type R1000-transformed readings.

*SP/hrpZ_Psph_*-expressing seedlings showed a considerable delay in symptom development in comparison with positive control plants. More specifically, all plants of this category developed very mild or no symptoms of chlorosis, whereas symptoms of stunting and necrosis were clearly absent in all these seedlings. ELISA test at 21 dpi, revealed that the transgenic root system of all 24 seedlings tested was virus-free or had virus titer close to the scoring threshold (average OD_405_ = 0.148). In contrast, the aerial non-transgenic parts of the same seedlings scored significantly higher virus titers (average OD_405_ = 0.492) than the root system (Table 3). Despite carrying considerable virus titers, the aerial parts of the *SP/hrpZ_Psph_*-expressing seedlings were either symptomless or presented mild symptoms (Figure 6).

## Discussion

In the present study, the transgenic expression of the harpin HrpZ_Psph_ from *P. syringae* pv. *phaseolicola*, has been deployed for a first time, as a means for evaluating the ability of the protein to elicit a general defense response which would result in protection against rhizomania, a serious disease of the sugar beet crop. Based on previous studies [39] indicating that stable transformants of *N. tabacum* expressing *hrpZ_Psph_* were phenotypically normal—did not show macroscopically visible necrosis even though they accumulated HR-active harpin—we constructed *N. benthamiana* transgenic lines expressing constitutively a fusion derivative (SP/HrpZ) with the signal peptide of the tobacco pathogenesis-related protein PR1a (SP-PR1a), as a means to direct harpin accumulation to the plant apoplast. Upon challenge with the virus, the *SP/hrpZ_Psph_*-expressing plants were highly resistant to BNYVV inoculation as evidenced by either a complete absence of disease symptoms or a considerable delay in symptom development. In addition, these phenotypic observations were accompanied by a significant reduction in virus multiplication, resulting in plants that were either completely virus free or had a very low virus titer even at 30 dpi. It is worth noting that transgenic plants expressing the canonical, non-secretable form of harpin (HrpZ_Psph_) were also made. However, these plants were not further analyzed because all the T0 transgenic lines (twenty two) shared similar phenotypic characteristics compared to non-transgenic plants regarding resistance to BNYVV inoculation and growth promotion (data not shown). Similarly, transgenic sugar beet hairy roots expressing the non-secretable form of harpin (HrpZ_Psph_) did not developed resistance to BNYVV inoculation (data not shown). All together, these data further support that the resistant phenotype is clearly correlated with the harpin targeted for secretion to the plant cell exterior. Our results corroborate earlier findings indicating on one hand an extracellular site of harpin action and on the other the ability of phytobacterial harpins to confer resistance to diverse pathogens [16], [18], [19], [28], [38]–[40], [54], [55]. Our findings therefore, are the first to reveal that expression of the SP/*hrpZ* gene from *P. syringae* pv. *phaseolicola* in transgenic *N. benthamiana* plants may confer high-level resistance against BNYVV.

Furthermore, the enhanced growth phenotype observed in *SP/hrpZ*-expressing plants of *N. benthamiana* has also been reported to occur upon external application of purified harpins from different phytopathogens [31], [35],[56],[57] as well as by transgene expression [29], [36], [58]. To the best of our knowledge, most harpin genes referred in the literature have been expressed *in planta* without a signal peptide. To this respect, the expression of a highly homologous harpin from *Pseudomonas syringae* pv. *syringae* [34] resulted in lack of plant growth promotion activity, whereas contradictory findings have been obtained by the expression of harpins from *E. amylovora* [29] and *Xanthomonas* species [29], [58]. The difference in the phenotypes developed by various harpins in transgenic plants may be due to the differences in their primary sequences and/or to the receptors present in the plant species tested.

We also investigated whether the virus resistance developed by harpin-expressing plants is associated with an induction of various defense-related genes. It is interesting that some defense genes (*AOX*, *NPR1*, *PR1a*) were slightly up-regulated in transgenic plants expressing either the secretable or non-secretable form of harpin suggesting that these genes might not be involved in harpin-induced resistance against BNYVV. On the other hand, the transcripts of two genes (*WIPK* and *hin1*) were more elevated in *SP-hrpZ_Psph_*-expressing plants. The higher expression level of the two latter defense-related genes suggests that the T1 *SP-hrpZ_Psph_* resistant plants display an enhanced defense state at the molecular level. This physiological condition may be similar to the so-called “primed state” [59]. The primed plants are often able to mount defense responses faster and stronger to biotic or abiotic stresses. It is worth noting that a priming mechanism could explain the enhanced resistance phenotype of transgenic cotton plants expressing harpin (*hpa_Xoo_*) against the fungal pathogen *Verticillium dahliae* [60]. In the future it will be important to investigate the transcriptomes of the transgenic plants expressing the secretable and the non- secretable form of harpin in order to unravel the signaling networks associated with the enhanced resistant state.

The priming could also provide a plausible explanation for the observed localized necrosis developed in the *SP*/*hrpZ_Psph_*-expressing leaves in the virus inoculation area. This necrosis was highly reproducible and observed only in resistant *SP/hrpZ* plants, while no visible necrosis was ever seen in the inoculated leaf area of non-transgenic or *hrpZ*-expressing plants [53]. It is tempting to speculate that extracellularly targeted harpin pre-activates cell death signaling pathway(s) and this activation could augment the defense responses upon virus infection, finally resulting in a macroscopically visible necrosis. Alternatively, the combined action of the endogenously expressed harpin and viral infection might activate different steps of defense responses and their synergistic effect ultimately leads to necrosis development at the site of infection. In either case, the local necrosis may prevent further virus spread. It is worth noting that a similar phenomenon was observed in transgenic tobacco plants expressing HrpZ*_Pss_* [34] where HR-like local lesions were observed in the lower leaves of transgenic plants following inoculation with the powdery mildew fungus, *Erysiphe cichoracearum*. Whereas this harpin was expressed without a signal peptide, the authors ascribed the necrotic phenotype to leakage of intracellularly accumulated HrpZ*_Pss_* from the *E. cichoracearum-*penetrated plant cells, allowing the protein to access the plant cell exterior where it normally acts, thus activating plant defense mechanisms. Further supporting such a hypothesis is the finding that simultaneous treatment of *A. thaliana* suspension cells with two different harpins, HrpN_Ea_ and HrpW_Ea_, induced a stronger cell death than the cell death observed in response to each harpin supplied separately [61]. The molecular mechanisms underlying the effects of harpin expression on BNYVV resistance and plant growth are needed to clarify by investigating the alterations occurring at the regulation of genes involved not only in the disease resistance but also in many metabolic pathways. Furthermore, the transcriptional changes in defense related genes upon virus infection will shed light on whether the disease resistance phenotype of transgenic plants is the effect of a priming mechanism.

The ability of the transgenically expressed SP/HrpZ protein to confer resistance to BNYVV was further confirmed in the sugar beet host by using an *A. rhizogenes*-based hairy root approach. Our findings demonstrate that expression of harpin in the sugar beet hairy root system results in absence of virus titer, indicative of high level resistance to BNYVV. Even, the aerial non-transgenic parts of the same seedlings were susceptible to BNYVV infection, though very mild or no symptoms were developed. Together the data clearly indicate the suitability of the methodology employed in infecting sugar beet seedlings and assessing BNYVV root resistance and further support the conclusion that the transgenic expression of SP/HrpZ_Psph_ through *A. rhizogenes*-mediated transformation may confer high level of BNYVV resistance to the root system of the sugar beet host.

In conclusion, our findings reinforce the suggestion that phytobacterial harpins may offer new opportunities for generating broad-spectrum resistance in plants. In particular, the data presented here provide evidence that the endogenously produced SP/HrpZ*_Psph_* may be employed for achieving high levels of resistance to the BNYVV in a model and a crop plant. While *N. benthamiana* provides a more amenable model system to elucidate the molecular mechanisms underlying SP/HrpZ-mediated BNYVV resistance, the creation of stable transformants of sugar beet, the natural host of BNYVV, might prove an efficient strategy to combat rhizomania. More importantly, the combined antiviral and plant growth enhancement effects of this protein, if also achievable in sugar beet, could prove valuable from the standpoint of crop's potential to become an important energy crop for the EU. Considering such an exploitation towards bio-economy products, such as bioethanol and biogas, and in view of no significant difficulties encountered in achieving an economically sound co-existence in sugar beet [62], the future perspective of deploying rhizomania resistant transgenic varieties should be expected as a promising strategy in complementing and enriching the breeder's arsenal. Such a probable development is in line with discussions which are underway in order to streamline the approval process for GM crops intended for purposes other than food and livestock feed [63].
